# A Decade’s Perspective on the Orthopedic Workforce in Saudi Arabia

**DOI:** 10.7759/cureus.37426

**Published:** 2023-04-11

**Authors:** Ahmed H AlHussain, Alwaleed A Alshahir, Abdullah Alhejji, Musaad M Bin Dukhi, Amjad AlGhamdi, Mohammed A Alfurayh, Nouf A Almagushi, Abdullah Bin Shabib, Abdulaziz M Bin Akrish

**Affiliations:** 1 Orthopaedic Surgery, King Abdulaziz Medical City, Riyadh, SAU; 2 College of Medicine, King Saud Bin Abdulaziz University for Health Sciences, Riyadh, SAU; 3 Medical Research, King Abdullah International Medical Research Center, Riyadh, SAU; 4 College of Medicine, Princess Nourah bint Abdulrahman University, Riyadh, SAU

**Keywords:** orthopedic surgeon, orthopedics, saudi arabia, orthopedic workforce, orthopedic surgery

## Abstract

Background

The orthopedic surgery workforce constitutes a vital role in the healthcare system, with data being scarce. Therefore, through this study, we share an overview of the orthopedic workforce distribution, demographic trends, and changes over the past decade in Saudi Arabia.

Methods

All practicing orthopedic surgeons in Saudi Arabia from January 1, 2010, to December 31, 2021, were included in the study. Data regarding orthopedic surgeons’ demographics and numbers were obtained from the Saudi Commission for Health Specialties (SCFHS), whereas the data related to the geographical distribution of orthopedic surgeons was obtained from the Ministry of Health Statistical Yearbook of 2020.

Results

The ratio of orthopedic surgeons per 100,000 people was 5.42 in 2010, which grew subsequently to 12.29 in 2021. The number of Saudi orthopedic surgeons has been noticeably rising through the years, while a slowly growing pattern can be seen among non-Saudi orthopedic surgeons. In addition, the highest ratios of orthopedic surgeons per 100,000 were in Makkah (1.72), Riyadh (1.26), and the Eastern Region (1.06).

Conclusion

In this study, we demonstrate the progress of the orthopedic workforce in Saudi Arabia over a period of 12 years. The number of orthopedic surgeons per 100,000 people showed a significant rise due to several factors, one of which is road traffic accidents. Also, although the number of female orthopedic surgeons has been rising lately, they are still much fewer than males in this field. In addition, Saudi Arabia has been developing a new healthcare system via the privatization of some of the governmental hospitals, which will lead to changes in the future workforce and its accommodations.

## Introduction

One of the key goals of Saudi Arabia's 2030 vision is to improve healthcare to fit the population's future needs by rising its number and gender distribution [1]. Orthopedic surgeons treat musculoskeletal diseases ranging from chronic to acute and life-threatening conditions. As a result, the orthopedic workforce is important in healthcare, and demand for such a workforce is predicted to rise as the population ages [2,3].

According to a 2014 study conducted in the United Kingdom, it is difficult to match the demand for orthopedic care with the number of surgeons available; thus, the British Ortho Association aimed to provide one physician for every 15,000 people by 2020 [4]. A more recent study predicted that the number of orthopedic surgeons working in rural areas would decline in the coming years. As a result of the labor misdistribution, these localities can expect inadequate access to orthopedic care [5]. Another study in the United States discovered that 25% of rural hospitals that participated in their survey did not provide any orthopedic surgical treatments, depriving people of much-needed services [6].

Despite the availability of sufficient data in other parts of the world, knowledge about the orthopedic workforce is severely deficient in Saudi Arabia. It is critical to assess if orthopedic training programs are enough to meet population demands, as well as their geographic and gender distribution across the country.

As a result, we undertook this study to assess future work-related challenges by analyzing Saudi Arabia's present orthopedic workforce demographics and developments over 12 years. The findings of this study are expected to have a substantial impact since they will expand on potential future issues for both beginning and experienced orthopedic surgeons. Furthermore, using the findings of this study will provide a clearer image of the workforce and supply of orthopedic surgeons in Saudi Arabia, giving the authorities meaningful data with which to act as necessary.

## Materials and methods

This study was on orthopedic surgeons working in Saudi Arabia from January 1, 2010, to December 31, 2021. Saudi Arabia is the fifth-largest nation in Asia and is divided into 13 regions. The General Authority for Statistics, Saudi Arabia, has estimated that Saudi Arabia's population is 34,218.169, as per the most recent estimate for 2019 [7].

The Saudi Commission for Health Specialties (SCFHS) was contacted via email to provide the following information in order to carry out the study's objectives: (1) the total number of orthopedic surgeons currently working in Saudi Arabia; (2) the number of orthopedic surgeons practicing in the country by year January 1, 2010, to December 31, 2021; (3) the number of Saudi and non-Saudi orthopedic surgeons currently working in Saudi Arabia; and (4) the number of male and female orthopedic surgeons. Also, information from the Ministry of Health's statistical yearbook for 2020 was used to determine the regional distribution of orthopedic surgeons who are currently working in the nation [8].

The King Abdullah International Research Center, National Guard Health Affairs, Riyadh, Saudi Arabia, approved this study (approval number: IRB/1903/22). Data entry and processing were conducted using Microsoft Excel 2016 (Microsoft Corporation, Redmond, Washington, United States). To calculate the ratio of orthopedic surgeons to the population, the number of orthopedic surgeons was divided by the entire population and then multiplied by 100,000.

## Results

As of June 2022, there were 3489 registered orthopedic surgeons in Saudi Arabia, with an estimated ratio of 12.29 orthopedic surgeons per 100,000 people. A large proportion of those orthopedic surgeons were ranked as registrars (35.71%, n=1246), and 96% of them were non-Saudis. In addition, the number of orthopedic surgeons ranked as consultants was 25.96% (n=906), with a proximal distribution between Saudis and non-Saudis. Out of the 557 training residents, only 10 were non-Saudis. The number of male residents was four times higher than the number of female residents. Moreover, there were three training residents for every five consultants. Further details regarding orthopedic surgeons' rank and their distribution are given in Table 1.

**Table 1:** Number of registered orthopedic surgeons by rank (as of June 2022)

Rank	Non-Saudi		Saudi		Total (%)
	Female	Male	Female	Male	
Consultant	1	425	12	468	906 (25.96)
Senior Registrar	5	367	24	171	567 (16.25)
Registrar	7	1189	5	45	1246 (35.71)
Training Resident	2	8	99	448	557 (15.96)
Resident	3	203	0	7	213 (6.10)
Total	18	2192	140	1139	3489

**Figure 1:**
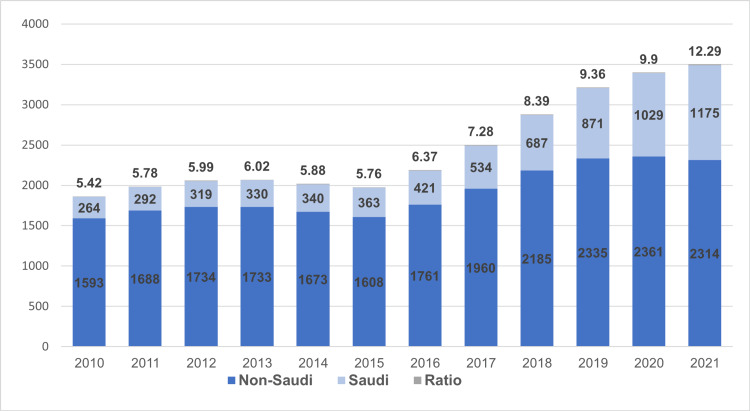
Saudi to non-Saudi orthopedic surgeons by year and ratio to 100,000 population.

Figure 1 shows the distribution of Saudi and non-Saudi orthopedic surgeons in Saudi Arabia for the 12 years of the study period. The ratio of orthopedic surgeons per 100,000 people was 5.42 in 2010, which grew subsequently to 12.29 in 2021. The number of Saudi orthopedic surgeons has been noticeably rising throughout the past few years in Saudi Arabia; on the other hand, a slowly rising pattern can be seen among non-Saudi orthopedic surgeons. Among Saudi orthopedic surgeons, the ratio per 100,000 people grew from the lowest point of 0.77 in 2010 to its highest point of 3.73 in 2021, while among non-Saudi orthopedic surgeons, the lowest ratio was 4.65 in 2010 and the highest was 6.89 in 2020.

**Figure 2:**
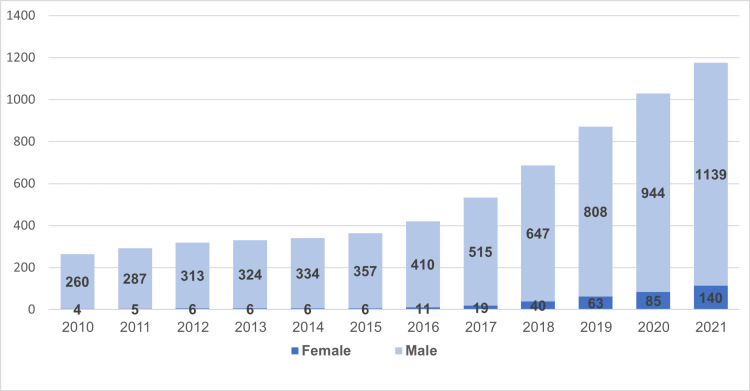
Number of Saudi male and female orthopedic surgeons by year.

Figure 2 shows the number of male and female Saudi orthopedic surgeons. In 2010, there were only four Saudi female orthopedic surgeons; however, this number grew to 140 in the year 2021. During the 12 years of the study period, the number of Saudi male orthopedic surgeons has raised from 260 in 2010 to 1139 in 2021.

**Figure 3:**
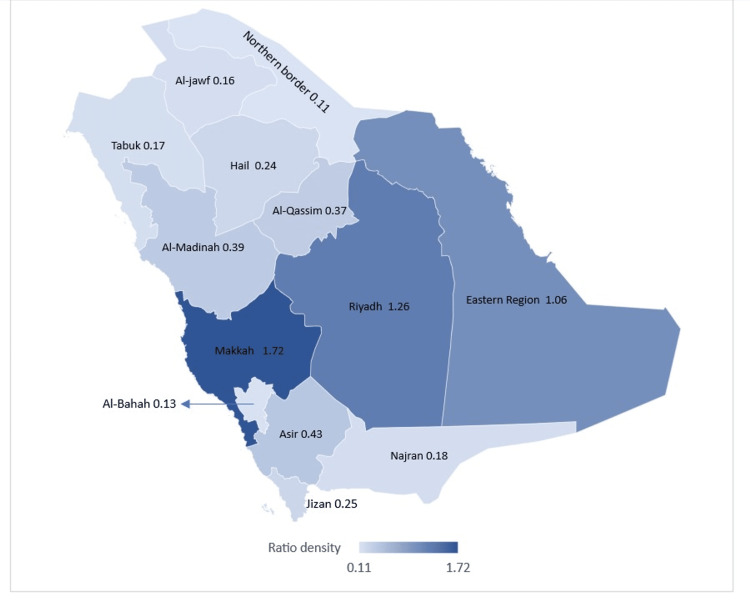
Orthopedic surgeons per 100,000 people in Saudi Arabia, 2020. The map was created with the help of Microsoft Excel and Bing Maps (Microsoft Corporation, Redmond, Washington, United States)

When calculating the number of practicing orthopedic surgeons in Saudi Arabia, 63.62% (n=2220) occupied the private and Ministry of Health (MOH) sectors. The remaining were in other governmental sectors such as the National Guard Health Affairs and security forces. The geographic distribution of orthopedic surgeons practicing in the private and MOH sectors is shown in Figure 3 per 100,000 people in 2020. the ratio per 100,000 people ranged between 0.11 on the Northern Border and 1.72 in Makkah. The highest ratios of orthopedic surgeons per 100,000 were in Makkah, Riyadh, and the Eastern Region, while the lowest were in the Northern Border, Al-Bahah, and Al-Jawf.

## Discussion

This is the first study to describe Saudi Arabia's present orthopedic workforce distribution, demographic trends, and developments over a decade. According to the findings of this study, the number of orthopedic surgeons has increased over the 12 years of the study period, with an estimated ratio of 12.29 orthopedic surgeons per 100,000 people. This rise could be due to a number of things, one of which is the burden of road traffic accidents (RTAs). RTAs are the primary reason for all trauma admissions to hospitals around the world [9].

Despite protective measures and programs, RTA is one of the leading causes of morbidity and mortality in Saudi Arabia [10]. Furthermore, over the last 20 years, Saudi Arabia has documented 611,000 RTA injuries, with 7% suffering from lasting disability [9]. Additionally, a Saudi Arabian study found that musculoskeletal injuries (95.5%) outnumbered central nervous system injuries (72.7%) in serious RTAs [11].

In light of all of these RTA injuries, local orthopedic surgeons are in high demand. The raised obesity rate in Saudi Arabia may potentially also be leading to a rise in demand for orthopedic surgeons [7]. Obesity is linked to a higher incidence and progression of osteoarthritis in both weight-bearing joints like ankles, knees, and hips and non-weight-bearing joints like hand joints, as well as higher rates of joint replacements and surgical complications [2]. Furthermore, it has been speculated that the demand for orthopedic surgeons who address these patients will only rise in the coming years due to rising obesity rates among specific demographics, particularly the elderly [3]. Total hip arthroplasty (THA) and total knee arthroplasty (TKA) have been identified as viable surgical therapeutic options for the therapy of osteoarthritis, involving the replacement of deteriorating joints with synthetic components that allow pain-free motion [4]. A prior study in Saudi Arabia found that, despite public concern and rejection of techniques like THA and TKA, there has been a surge in TKA cases in recent years, with excellent improvements in knee function and pain reduction [5]. Many of these causes have contributed to the raised need for orthopedic surgeons in Saudi Arabia.

In 2017, over 22.3 million orthopedic surgical procedures were performed worldwide [6]. The number of procedures performed per year is predicted to increase at a compound annual rate of 4.9% between 2017 and 2022, approaching 28.3 million by 2022 [6]. Because of this tremendous expansion, a greater number of orthopedic surgeons are required to avoid a physician shortage. According to the American Academy of Orthopedic Surgeons (AAOS), they addressed the trends in orthopedic surgery in the United States and found that there has been a constant growth in the number of physicians in orthopedic practices during a 10-year period between 2008 and 2018 [8].

Furthermore, according to the AAOS data, the density of orthopedic surgeons in 2018 was 9.3 per 100,000 people [12]. Even so, the Association of American Medical Colleges (AAMC) predicts a physician shortfall of up to 122,000 by 2032 [13]. According to a Chilean study, there will be 1770 orthopedic surgeons registered in the National Superintendence of Health's registry in 2020, which is 1.6 times the number in 2004. Furthermore, the predicted density of orthopedic doctors in Chile for 2020 was found to be 8.6 per 100,000 persons [14]. Furthermore, the British Orthopedic Association has planned to deliver a consultant-to-population ratio of 1:15,000 by 2020, which is a rise from prior years [15].

The number of orthopedic surgeons globally has grown over time because of factors such as the increased occurrence of RTA, obesity, and population aging. This rapid development in the number of orthopedic surgeons worldwide corresponds with the findings of this study, which indicated that the number of Saudi orthopedic doctors has been steadily expanding in recent years.

Throughout the last few decades, the proportion of women entering the medical field has steadily raised; currently, they account for roughly half of the medical schools [3]. As a result, more women are pursuing careers in surgical disciplines [2]. Orthopedics, on the other hand, continues to recruit female medical students at a rate that trails all other surgical specialties. A lack of effective mentorship was cited by 69% of women in a poll done in the United States as a reason for not choosing orthopedic surgery [13]. Mentoring that supports and promotes gender diversity can thereby increase medical students' interest in orthopedic surgery and, as a result, increase the number of female orthopedic surgeons [16].

Female surgeons can help reduce healthcare disparity and provide culturally competent care, thus governments all around the world, including Saudi Arabia, are attempting to close the gender gap in orthopedics [17,18]. The number of orthopedic training programs for women in the United States has expanded significantly during the first five years, 2004-2009, and the most recent, 2014-2019 [16]. Females make up more than 57% of certified medical residents in the United States. Yet less than half of those who applied opted for a surgical residency [2]. According to Blakemore et al., the proportion of female orthopedic residents has raised from 0.6% in 1970 to roughly 50% today, compared to approximately 9% in 2001 [19].

In the United Kingdom, medical applications in 2020 revealed that 55% of applicants were female, with less than 30% continuing to higher surgical training [6]. Nevertheless, females constituted less than 25% of UK orthopedic trainees and fellows in 2020 and only 7% of orthopedic consultants [7]. According to Chilean research, the proportion of female orthopedic surgeons was low from 2004 to 2020, peaking at 7.6% in 2020 [13]. A cross-sectional study in Saudi Arabia revealed that the gender distribution of females in orthopedic surgery was 14.6% (37), compared with 85.4% (217) for males [20].

Nevertheless, the percentage of female orthopedic surgeons in Saudi Arabia raised at a compound annual growth rate of 2% between 2010 and 2019. This study also shows a rise in the number of female orthopedic surgeons, with the number of Saudi female orthopedic surgeons increasing from 4 in 2010** to 115 in 2021.**

Saudi Arabia has 3489 orthopedic surgeons working in both government and private facilities in 2021. Just 1175 of those orthopedic surgeons were Saudi, with the remainder being non-Saudi. Saudi physicians are in short supply due to the country's ever-increasing need for healthcare. As a result, the number of foreign healthcare personnel in the country is rising. Their position is critical for ensuring continuity of care and providing the highest quality of treatment to patients. However, relying on foreign healthcare staff has significant drawbacks. They have a higher turnover rate and leave abruptly, which contributes to a significant financial cost in terms of training and employing other physicians in their place [21]. Furthermore, language difficulties are a major source of misunderstanding between patients and medical staff, lowering the standard of service and decreasing patient satisfaction [1]. In a survey of 16 physicians, the majority of whom were non-Arabic, communication difficulties were reported in the first two to three years [2].

Regional maldistribution of orthopedic surgeons is an issue for the global healthcare system, and Saudi Arabia is no exception. Orthopedic physicians in Saudi Arabia prefer to practice in cities rather than rural areas. According to SCFHS data, Makkah region has the most orthopedic surgeons, with 587, followed by Riyadh, which has 432 orthopedic surgeons. This might be attributed to increasing work opportunities as well as the modern lifestyle provided by large cities.

In the United States, there is also a considerable disparity in the number of orthopedic surgeons in urban versus rural areas. According to a study of 225 rural hospitals in the United States, just 30% had at least one full-time orthopedic surgeon. As a result, residents in such communities must drive an average of 55 miles for orthopedic emergencies, which may result in the patient's condition deteriorating before receiving medical care [4].

Because of the establishment of the 2030 vision, Saudi Arabia is set to undergo massive transformations and developments during the next 10 years. The government-private partnership is one of the projects aiming to improve healthcare. Saudi Arabia plans to privatize 290 hospitals and 2,900 primary care clinics by 2030, resulting in more comprehensive medical insurance for all Saudi nationals [5,6]. Furthermore, the government's public-private partnership will allow residents to select from a variety of healthcare providers. Additionally, Saudi Arabia has encouraged and welcomed full-ownership foreign investments in the healthcare industry [6].

Our research uncovered the Saudi orthopedics workforce from 2010 to 2021. The study, however, had several drawbacks. There is a dearth of data on orthopedic surgeons' working hours, ages, and workloads. The preceding variables may be useful in determining the shortage and surplus of orthopedic surgeons in Saudi Arabia. Although other studies have assessed workforce requirements, we believe they are not appropriate for our study for various reasons. Secondly, there is a shortage of data on physicians leaving their jobs due to death or retirement. Furthermore, the Saudi Arabian government intends to privatize health care and provide health insurance to all people. As a result, patients will have the option of continuing to work with their healthcare provider. The burden, we feel, will be passed to the private sector. To compensate for the rising load, the private sector would either raise working hours or hire more healthcare staff [22,23]. Another restriction was a lack of data from the military sectors for the number of orthopedic surgeons, which may have produced a more accurate estimate of the orthopedic surgeons' ratio per 100,000 persons.

## Conclusions

We demonstrated the progress of the orthopedic workforce in Saudi Arabia over a period of 12 years. The number of orthopedic surgeons per 1,000 people showed a significant rise due to several factors, one of which is RTA. Furthermore, a higher ratio of Saudi orthopedic surgeons has been observed compared to orthopedic surgeons of other nationalities. Although the number of female orthopedic surgeons has recently raised, they remain far fewer than males in this specialty. Geographical maldistribution is one of the challenges that many countries, including Saudi Arabia, face, and it may have an impact on the quality of healthcare offered. Saudi Arabia has been constructing a new healthcare system through the privatization of several state institutions, which will result in changes to the future workforce.
